# Cloning and expression of P67 protein of *Mycoplasma leachii*

**DOI:** 10.14202/vetworld.2017.1108-1113

**Published:** 2017-09-21

**Authors:** Sabarinath Thankappan, Rajneesh Rana, Arun Thachappully Remesh, Valsala Rekha, Viswas Konasagara Nagaleekar, Bhavani Puvvala

**Affiliations:** Division of Bacteriology and Mycology, Indian Veterinary Research Institute, Izatnagar, Uttar Pradesh, India

**Keywords:** cloning, dot blot, expression, *Mycoplasma leachii*, P67 protein, western blot

## Abstract

**Aim:**

The present study was undertaken to clone, express and study the immunogenicity of P67 protein of *Mycoplasma leachii*.

**Materials and Methods:**

P67 gene was amplified from genomic DNA of *M. leachii*. The polymerase chain reaction (PCR) product was inserted in pRham N-His SUMO Kan vector and was used to transform competent *Escherichia cloni* 10G cells. Recombinant protein expression was done by inducing cells with 0.2% Rhamnose. Purification was done using nickel nitrilotriacetic acid affinity chromatography. Western blot and dot blot analysis were performed to assess the immunoreactivity of P67 protein.

**Results:**

PCR amplicon size of P67 gene was found to be 1500 base pair. The size of the fusion protein with SUMO tag was 79 kDa in sodium dodecyl sulfate polyacrylamide gel electrophoresis analysis. The recombinant P67 fusion protein expressed in pRham N-His SUMO Kan vector was found to be immunogenic in both western blot and dot blot analysis.

**Conclusion:**

Western blot and dot blot analysis of P67 protein of *M. leachii* revealed that the protein is immunogenic. Further work is needed to evaluate the role of P67 antigen of *M. leachii* as an immunodiagnostic agent.

## Introduction

*Mycoplasma* sp. bovine group 7 (MBG7) has been implicated as a causative agent in severe outbreaks of mastitis, polyarthritis, pneumonia, and abortion in dairy cattle [1,2]. The type strain of MBG7 is PG50, previously known as N29, was first isolated from joint fluids of arthritic calves [3]. MBG7 has been described as a chimera between *Mycoplasma capricolum* and *Mycoplasma mycoides* [4,5], and group of MBG7 strains remained unassigned [6]. However, MBG7 has been assignment as a separate species, namely, *Mycoplasma leachii* sp. nov., since it is placed at an intermediary position between *M. mycoides* and *M. capricolum* as assessed by DNA–DNA hybridization and protein patterns [7]. Thus, *M. leachii* is included as one of the five species in “*M. mycoides* cluster” [8,9].

*M. leachii* is common and potentially distributed worldwide with a wider geographic presence [10]. In Australia, an outbreak of bovine mastitis, polyarthritis, and pneumonia due to *M. leachii* infection was reported in which the organism was isolated in heavy pure growth from joint fibrin, synovial membranes and lungs [11]. Apart from Australia, isolation of *M. leachii* strains has been reported from urogenital tract of cattle and aborted fetuses in the United States [12]. In Canada, *M. leachii* has been isolated from the reproductive tract of cattle as well as from the stomach contents and internal organs of aborted fetuses [13]. In China, severe economic losses due to polyarthritis caused by *M. leachii* in 350 female calves resulted in high mortality and the recovered calves being culled due to the permanent disfigurement of the appendicular skeleton [10]. In India, *M. leachii* was isolated from the uterine discharge of aborting buffaloes and from preputial washings of Buffalo Bulls [14,15].

Even though *M. leachii* has been isolated from both healthy cattle [1,12] as well as from small ruminant hosts [16], *M. leachii* is capable of multiplying within different tissue sites within host species and among different animals within a herd and these properties make *M. leachii* a virulent, invasive organism able to cause systemic infection. Moreover, genetic fingerprinting studies of 24 isolates which were indistinguishable from one another recovered from multiple tissue sites and body fluids of infected calves suggested that *M. leachii* was capable of systemic infection with significant economic losses to dairy operations [17]. *M. leachii* is unique among bacteria as it possesses linear β (1→2)-glucopyranose homopolymer in both its capsular polysaccharide and exopolysaccharide which is rarely found in other bacteria and is responsible for induction of exacerbated lung inflammation [18]. The ability of *M. leachii* to produce arthritis was confirmed following the intravenous inoculation of a culture of this organism [19]. Epidemiologic and clinical investigations of *M. leachii* point toward the use of contaminated semen as a source of infection for artificially inseminated cows [20]. The great affinity of *M. leachii* for synovial epithelium, the septicemic and fibrinopurulent nature of infection and the poor response to anti-mycoplasmal drugs and often permanent articular disfigurement in calves after recovery makes this disease a severe blight for dairy industry [10].

Apart from a few commercially available whole cell antigen based enzyme-linked immunosorbent assay (ELISA), there is a dearth in the availability of immunodiagnostics of high sensitivity and specificity such as recombinant protein based diagnostic assay for early and rapid detection of antibodies against *M. leachii* infection. However, the complete genome sequence of *M. leachii* strain PG50 is currently available [21], which revealed a total of 1732 proteins for *M. leachii*. Out of 1732 proteins, a lipoprotein of 532 amino acids having a predicted molecular weight of 61.181 kDa as per analysis using Uniprot software was selected. This lipoprotein which contained no internal in frame UGA_Tryptophan_ (UGA_Trp_) codons was designated as P67 based on the observed molecular weight obtained in sodium dodecyl sulfate polyacrylamide gel electrophoresis (SDS-PAGE). The expression of recombinant proteins of *Mycoplasma* in heterologous hosts such as *Escherichia cloni* 10G cells is hampered by the presence of internal in frame UGA_Trp_ codons which code for amino acid tryptophan in mycoplasmas but is considered as stop codon in *E. cloni* 10G cells [22]. Hence, P67 was selected as it was found to be the most promising candidate for full-length expression in heterologous host such as *E. cloni* 10G cells.

Moreover, the detailed analysis of P67 lipoprotein using B-cell epitope prediction software such as BepiPred 1.0 server [23], revealed four linear B-cell epitope regions from amino acid positions 33-87 (55 amino acids), 94-118 (25 amino acids), 185-201 (17 amino acids), and 305-316 (12 amino acids). The findings of BepiPred 1.0 server, which predicts the location of linear B-cell epitopes using a combination of a hidden Markov model and a propensity scale method, was further supported by BcePred server [24]. According to BcePred server which predicts B-cell epitopes based on physiochemical properties of amino acids such as hydrophilicity, flexibility/mobility, accessibility, polarity, exposed surface, and turns, there were four stretches of amino acid residues acting as epitopes in P67 lipoprotein at exactly the same position predicted by BepiPred 1.0 server. Hence, the present study was undertaken to assess the immunogenic potential of P67 antigen of *M. leachii* by western blot and dot blot analysis so that it can be exploited for the development of recombinant antigen based diagnostic test for detection of *M. leachii* in bovines. The identification and characterization of important immunogens of *M. leachii* play a pivotal role in the designing and development of diagnostics against *M. leachii* since isolation of the organism is rarely reported [25].

## Materials and Methods

### Ethical approval

The Institutional Animal Ethics Committee/Committee for the Purpose of Control and Supervision of Experiments on Animals of the Indian Veterinary Research Institute (IVRI), Izatnagar, had approved for immunization studies.

### Sonicated antigen preparation of M. leachii strain PG50 for raising hyper immune sera

Sonicated antigen of *M. leachii* strain PG50 for raising the antiserum was prepared as per protocol of Bhanuprakash and Srivastava, 1996 [26] with some modifications. The colonies of *M. leachii* strain PG50 were cut out from solid medium and transferred to 5 ml of modified Pleuropneumonida like organim (PPLO) broth medium, incubated for 48 h at 37°C. It was then transferred to 50 ml medium and incubated for 48 h and finally, 50 ml were transferred further to 500 ml of medium and incubated in shaker incubator for 48 h. Cells were harvested by centrifugation at 10,000 rpm at 4°C for 30 min and washed 3 times in phosphate-buffered saline (PBS) pH 7.4. The cell pellet was resuspended in 3 ml sterile PBS and treated ultrasonically (MSE Soniprep150, USA) at 10 µ by applying 10 jerks, each of 60 s with a gap of 60 s in an ice jacket. Protein concentration of antigen was estimated by Bradford method, and protein concentration was standardized to 4 mg/ml using sterile PBS.

### Raising of hyper immune sera against whole cell antigen of M. leachii in calves

Hyper immune sera against whole cell antigen of *M. leachii* in calves was prepared as per protocol of Bhanuprakash and Srivastava, 1996 [26] with some modifications. Two bull calves of age 6 months which were procured from Livestock Production and Management section, IVRI were used for raising hyper immune sera against sonicated whole cell antigen of *M. leachii* strain PG50. Before preparation of hyper immune sera, both the animals were bled from jugular vein, and sera were tested for the presence of antibodies against whole cell antigen of *M. leachii* by both double immunodiffusion (DID) and indirect hemagglutination test (IHA). Once the sera were confirmed as negative for the presence of antibodies against whole cell antigen of *M. leachii*, the negative sera were stored at −20°C for further use. The animals were injected subcutaneously with an emulsion consisting of 2.5 ml of sonicated antigen of *M. leachii* mixed with 2.5 ml of Freund’s complete adjuvant. Booster injections were given at 14^th^, 21^st^, 28^th^, and 35^th^ days with 1.25 ml of antigen mixed with 1.25 ml of Freund’s incomplete adjuvant. Blood was collected 7 days after injection of 4^th^ booster antigen, and serum was stored at −20°C for further use.

### Extraction of total genomic DNA

Genomic DNA was extracted from culture of *M. leachii* maintained in the Division of Bacteriology and Mycology, IVRI, Izatnagar, by cetyltrimethylammonium bromide method and was preserved for further use.

### Polymerase chain reaction (PCR) amplification and cloning of P67 gene of M. leachii

PCR amplification of P67 gene was performed using primers (forward primer: 5’- CGCGAACAGATTGGAGGTAAACAACCAGATAAAAAACCT-3’) and (reverse primer: 5’- GTGGCGGCCGCTCTATTATTGAATTAAAGCGGTTTGTAC-3’) to amplify 1500 base pair (bp) amplicon from *M. leachii* genomic DNA. Primers were designed taking the reference sequence available at GenBank (AAC06131.1). 18 nucleotides at the start of both the primers (underlined) were introduced to facilitate cloning into pRham N-His SUMO expression vector by homologous recombination. The PCR was carried out in 25 µl reaction volume containing 1 µl of each primer (10 picomol/µl), 0.2 mM of deoxynucleotide triphosphates, 1.5 mM of MgCl_2_, 1 unit of vent DNA polymerase (New England Biolabs, UK) in 1× reaction buffer using 50 ng of genomic DNA as a template. The reaction conditions were as follows: Initial denaturation 95°C for 5 min, 35 cycles of denaturation at 94°C for 1 min, annealing at 60°C for 1 min, and extension at 72°C for 1.5 min followed by final extension at 72°C for 10 min. The amplified products were analyzed by electrophoresis in 1.5% agarose containing ethidium bromide (0.5 µg/ml). The amplicon was inserted into pRham N-His SUMO expression vector. The newly constructed recombinant plasmid was designated as p67 vector and was transformed into competent *E. cloni* 10G cells. Recombinants obtained on Luria-Bertani (LB) Kanamycin agar containing 30 µg/mL kanamycin were screened through colony PCR.

### Recombinant protein expression and purification

*E. cloni* 10G cells harboring p67 vector were grown in LB medium till the optical density 600 nm reached 0.2-0.8. The cells were then induced with 0.2% Rhamnose and allowed to grow overnight at 37°C. Cells were harvested and the proteins analyzed by SDS-PAGE. The purification of the recombinant protein was done using nickel nitrilotriacetic acid (Ni-NTA) affinity chromatography. The recombinant P67 (rP67) protein present in inclusion bodies inside *E. cloni* 10G cells was solubilized using lysis buffer containing 6 M guanidinium hydrochloride at pH 8.0. The lysis buffer was passed twice through Ni-NTA affinity column which facilitated the binding of the His-tagged recombinant protein. Wash buffer (8 M urea; pH 6.3) was passed though the column to remove proteins of *E. cloni* 10G cells and the purified rP67 protein was eluted using elution buffer (8 M urea; pH 4.5), and purification efficiency was checked using SDS-PAGE.

### Western blot and dot blot analysis

Western blot was performed by separating the purified recombinant protein on 12% SDS-PAGE and transferring it onto nitrocellulose membrane (NCM) by applying current at 0.8 mA/cm^2^. After overnight blocking with 3% skimmed milk, P67 protein present on the membrane were then immune stained by first exposing the membrane to hyper immune sera raised against *M. leachii* in calves (positive control) and known negative bovine sera confirmed by both DID and IHA) at a dilution of 1:100 followed by species specific secondary antibodies conjugated to horseradish peroxidase at a dilution of 1:5000 and finally the reaction was developed with 4-chloro-1-naphthol (Sigma) and H_2_O_2_. Similarly, dot blot analysis was performed using hyper immune sera as a positive control and known negative sera as a negative control by following the protocol used in western blot after charging the NCM with purified P67 protein.

## Results and Discussion

**Figure-1:**
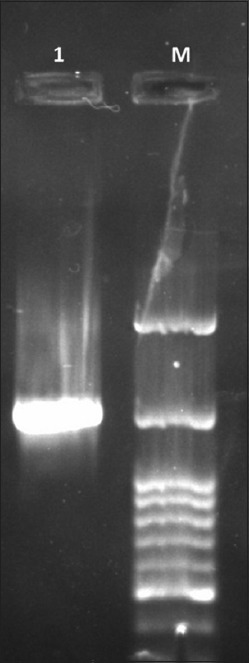
Polymerase chain reaction amplification of P67 gene of *Mycoplasma leachii*.

**Figure-2:**
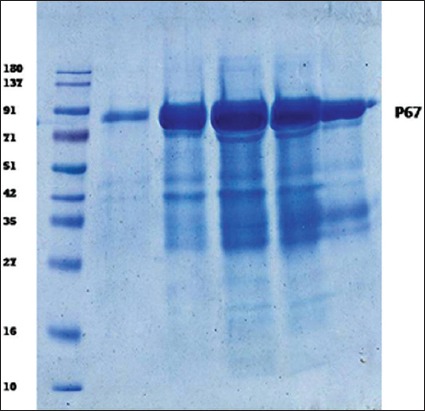
Sodium dodecyl sulfate-polyacrylamide gel electrophoresis analysis of purified p67 protein.

**Figure-3:**
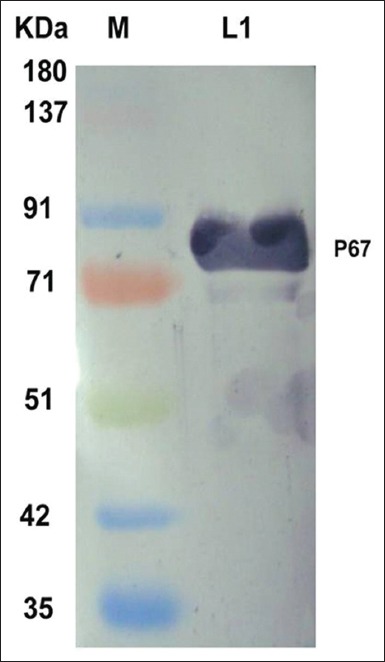
Western blot analysis of the purified p67 protein against hyper immune sera.

**Figure-4:**
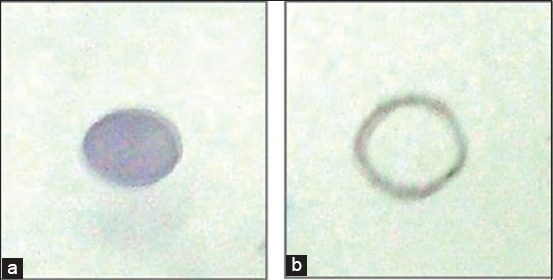
(a and b) Dot blot analysis of the purified p67 protein.

The 1500 bp amplicon size of PCR product visualized in agarose gel electrophoresis indicated that P67 gene amplification has taken place using *M. leachii* genomic DNA as template (Figure-1). The colonies obtained in LB Kanamycin agar were confirmed as transformants by colony PCR. rP67 protein expression was confirmed by SDS-PAGE analysis in which a 79 kDa rP67 protein was produced after induction with 0.2% Rhamnose (Figure-2). In this study, we obtained a high-level P67 gene expression of approximately 20 mg purified protein per liter of induced culture. Western blot and dot blot analysis of the purified P67 protein were performed using both hyper immune sera raised against whole cell antigen of *M. leachii* PG50 strain and negative sera confirmed by both DID and IHA. The negative sera did not react with rP67 protein in both western blot and dot blot analysis. However, the hyper immune sera reacted strongly with rP67 protein in both western blot and dot blot analysis which confirmed that the protein is highly immunogenic (Figures-3 and 4).

The diagnosis of *M. leachii* is difficult owing to the fact that isolation of MBG7 strains is tedious and hence only rarely described. This may be the reason that *M. leachii* is one of the least reported mycoplasmas to cause infection among the *M. mycoides* cluster [27]. P67 gene of *M. leachii* was used in this study since this gene was highly conserved in this species as shown by the analysis of strains isolated from different hosts and from geographically distant origins. Further, PCR amplification of P67 gene of *M. leachii* showed that the gene is conserved and expressed in all different strains of *M. leachii* tested, but not in related mycoplasmas [25]. One of the main drawbacks associated with expression of recombinant protein of mycoplasmas in heterologous hosts such as *E. cloni* 10G cells is the presence of internal in frame UGA_Trp_ codons which code for amino acid tryptophan in mycoplasmas but is considered as stop codon in *E. cloni* 10G cells. The DNA sequence of the P67 gene showed that it contained no internal in frame UGA_Trp_ codons and is, therefore, an attractive candidate for full-length expression in *E. cloni* 10G cells [22]. The complete genome sequence of *M. leachii* PG50 is available under accession number CP002108.1 which opens new vistas in identification of novel candidate antigens besides P67 suitable as immunodiagnostic for detection of *M. leachii* infection [21].

The main bottleneck involved in whole cell antigen based immunoassays for *M. leachii* is the preparation of whole cell antigen of *M. leachii* which is time-consuming and cumbersome. The advent of recombinant DNA technology made possible the generation of large quantities of purified recombinant proteins in heterologous hosts such as *E. cloni* for specific diagnosis of infectious diseases [28]. Moreover, recombinant protein based serological tests show high sensitivity and specificity, owing to high concentration of immunoreactive antigens and the lack of non-specific moieties present in whole cell preparations [29]. Thus, recombinant antigen based diagnostic tests have effectively replaced whole cell antigen based immunodiagnostic assays in the diagnosis of various *Mycoplasma* infections pertaining to veterinary field [30-32]. Moreover, several authors have recently claimed that recombinant antigen based immunodiagnostics, especially ELISA have proven to be highly sensitive and specific method for the early diagnosis of bovine mycoplasmas such as *M. bovis* infection [33-35]. Hence, rP67 antigen based diagnostic tests whose immunoreactivity is proven in this study by both western blot and dot blot is the appropriate antigen in the diagnosis of *M. leachii* infection.

## Conclusion

In this study, it was concluded on the basis of western blot and dot blot analysis that rP67 protein of *M. leachii* has immunogenic potential and is, therefore, a suitable candidate antigen in the development of immunodiagnostics.

## Authors’ Contributions

RR, VKN, and ST prepared the study design and carried out the experiment. ST, ATR, VR, and BP conducted the molecular part of the research. RR, VKN, and ST analyzed the data, drafted and revised the manuscript. All authors read and approved the final manuscript.
